# In Vivo 7-Tesla MRI Investigation of Brain Iron and Its Metabolic Correlates in Chronic Schizophrenia

**DOI:** 10.1038/s41537-022-00293-1

**Published:** 2022-10-26

**Authors:** Parsa Ravanfar, Warda T. Syeda, Mahesh Jayaram, R. Jarrett Rushmore, Bradford Moffat, Alexander P. Lin, Amanda E. Lyall, Antonia H. Merritt, Negin Yaghmaie, Liliana Laskaris, Sandra Luza, Carlos M. Opazo, Benny Liberg, M. Mallar Chakravarty, Gabriel A. Devenyi, Patricia Desmond, Vanessa L. Cropley, Nikos Makris, Martha E. Shenton, Ashley I. Bush, Dennis Velakoulis, Christos Pantelis

**Affiliations:** 1 Melbourne Neuropsychiatry Centre, Department of Psychiatry, The University of Melbourne and Melbourne Health, Carlton South, VIC Australia; 2 Psychiatry Neuroimaging Laboratory, Brigham and Women’s Hospital and Harvard Medical School, Boston, MA USA; 3 Department of Psychiatry, The University of Melbourne and Melbourne Health, Parkville, Australia; 4 Center for Morphometric Analysis (CMA), Massachusetts General Hospital, Charlestown, MA USA; 5 Department of Anatomy and Neurobiology, Boston University School of Medicine, Boston, MA USA; 6 Melbourne Brain Centre Imaging Unit, Department of Radiology, University of Melbourne, Parkville, VIC Australia; 7 Department of Radiology, Brigham and Women’s Hospital and Harvard Medical School, Boston, MA USA; 8 Department of Psychiatry, Brigham and Women’s Hospital, Harvard Medical School, Boston, MA USA; 9 Department of Psychiatry, Massachusetts General Hospital, Harvard Medical School, Boston, MA USA; 10 Department of Biomedical Engineering, The University of Melbourne, Parkville, VIC Australia; 11 Melbourne Dementia Research Centre, The Florey Institute of Neuroscience & Mental Health, and The University of Melbourne, Parkville, VIC Australia; 12 Centre for Psychiatry Research, Department of Clinical Neuroscience, Karolinska Institutet, Stockholm, Sweden; 13 Cerebral Imaging Center, Douglas Research Centre, Montreal, QC Canada; 14 Department of Psychiatry, McGill University, Montreal, QC Canada; 15 Department of Biomedical Engineering, McGill University, Montreal, QC Canada; 16 Department of Radiology, Royal Melbourne Hospital, University of Melbourne, Parkville, VIC Australia; 17 Neuropsychiatry, The Royal Melbourne Hospital, Parkville, VIC Australia; 18 The Florey Institute of Neuroscience and Mental Health, The University of Melbourne, Parkville, VIC Australia

**Keywords:** Schizophrenia, Molecular neuroscience

## Abstract

Brain iron is central to dopaminergic neurotransmission, a key component in schizophrenia pathology. Iron can also generate oxidative stress, which is one proposed mechanism for gray matter volume reduction in schizophrenia. The role of brain iron in schizophrenia and its potential link to oxidative stress has not been previously examined. In this study, we used 7-Tesla MRI quantitative susceptibility mapping (QSM), magnetic resonance spectroscopy (MRS), and structural T_1_ imaging in 12 individuals with chronic schizophrenia and 14 healthy age-matched controls. In schizophrenia, there were higher QSM values in bilateral putamen and higher concentrations of phosphocreatine and lactate in caudal anterior cingulate cortex (caCC). Network-based correlation analysis of QSM across corticostriatal pathways as well as the correlation between QSM, MRS, and volume, showed distinct patterns between groups. This study introduces increased iron in the putamen in schizophrenia in addition to network-wide disturbances of iron and metabolic status.

## Introduction

Schizophrenia is a debilitating neuropsychiatric disorder with a complex neuropathology that is not yet fully understood. Recent studies provide evidence of increased oxidative stress^1^, neuroinflammation^2^, activation of matrix metalloproteinase-9 (MMP-9)^3^, and mitochondrial dysfunction^4^ in schizophrenia. These interconnected pathologic processes share a common element, iron. Iron is an essential metal ion with crucial physiologic roles in the brain, such as oxidative metabolism, myelin production and neurotransmitter synthesis^5^. However, dysregulated iron becomes a catalytic generator of oxidative moieties, induces mitochondrial dysfunction^6^, activates MMP9^7^, and is associated with neuroinflammation^8^. Of further importance, iron is closely linked to dopaminergic neurotransmission, one of the main neurotransmitter systems that is shown to be abnormal in schizophrenia^9^. Iron is the cofactor of tyrosine hydroxylase, the key rate-limiting enzyme in the dopamine production pathway, and essential for dopamine synthesis^10^. Iron also has a regulatory effect on tyrosine hydroxylase activity, and can induce dopamine synthesis^11^, while dopamine has been observed to promote cellular uptake of iron^12^. In addition, iron is involved in the intracellular trafficking and membrane integration of D2 receptors, and iron chelation has been shown to impede post-synaptic dopaminergic neurotransmission^13^. In fact, the distribution of iron within the brain is heterogeneous, with the highest concentrations in the main dopaminergic brain regions, the substantia nigra, pallidum, and striatum^14^. Based on this link between iron, dopaminergic neurotransmission, and multiple pathologic processes that have been identified in schizophrenia, it is timely to study the role of iron in the pathology and neurochemistry of this disorder. Hyperactive dopamine neurotransmission is potentially associated with iron accumulation that can induce neurotoxicity and synaptotoxicity through oxidative stress and ferroptosis^15^, and lead to the progressive brain changes observed in schizophrenia^16,17^.

Observational studies using histological methods sparked interest in studying basal ganglia mineralization in individuals with schizophrenia (see Casanova et al., 1990). The first human studies investigating brain iron in schizophrenia were postmortem brain tissue examinations dating back to the early 1990s, with reports of higher iron content in the caudate nucleus^18^ and globus pallidus^19^, while another study found no changes in the caudate nucleus^20^. The limitations associated with postmortem brain tissue analysis have resulted in a paucity of literature on brain iron in schizophrenia. Nevertheless, recent neuroimaging developments have allowed for the in vivo measurement of iron using its paramagnetic characteristics. Quantitative susceptibility mapping (QSM) is an MRI technique that offers superior accuracy in the quantification of iron compared to other available tools^21^. QSM has been widely used to study brain iron concentrations in several neurodegenerative disorders (for review see Ravanfar et al., 2021) and has demonstrated constitutively higher iron levels in brain regions centrally associated with schizophrenia pathology^22^.

In the present study, using ultra-high field 7 T QSM and magnetic resonance spectroscopy (MRS), we aimed to evaluate brain iron, glutathione (GSH, a marker of oxidative stress) and lactate (a marker of anaerobic metabolism) in individuals with chronic schizophrenia. The analyses focused on cortico-subcortical pathways of the limbic and associative striatum based on the existing literature that describes aberrant dopaminergic activity within these pathways^9,23^. The cortico-subcortical pathways include the prefrontal and medial temporal cortices, striatum, thalamus, hippocampus, and substantia nigra. The prefrontal cortex and hippocampus have been found to display progressive changes across the illness spectrum^16,24–27^. We hypothesized that iron concentration would be increased in the dopaminergic subcortical structures along with a network-wide disruption of iron levels within the dopaminergic cortico-subcortical pathways. Further, we hypothesized that the dysregulation of iron would be associated with oxidative stress, impaired mitochondrial metabolism, and volume reduction in the cortical regions involved in the cortico-subcortical pathways.

## Results

### Demographic information, clinical assessments, and regional volumetric comparisons between groups

As demonstrated in Table 1, participants in control and schizophrenia groups did not differ in age, sex, body mass index and serum iron. Demographic information and clinical assessments are summarized and compared between groups in Table 1. Comparison of volume in the regions of interest (ROIs) between groups is provided in Supplementary Table 1 and Supplementary Fig. 1.

**Table 1:** Comparison of demographics, clinical assessments, and medications.

	*schizophrenia (n* = *12)*	*control (n* = *14)*	*p-value*
*age, years, mean (SD)*	36.2 (10.1)	32.6 (9.2)	0.64
*sex (M:F)*	7:5	6:8	0.63
*serum iron, µmol/L, mean (SD)*	17.1 (5.6)	23 (11)	0.12
*body mass index, mean (SD)*	27.1 (4.4)	24.7 (4.7)	0.23
*BPRS, mean (SD)*	43.5 (7.9)	14.8 (13.4)	<0.001
*SANS, mean (SD)*	37.9 (22.4)	2.5 (3.6)	<0.001
*SOFAS, mean (SD)*	67.2 (18.7)	90.7 (4.7)	<0.001
*MADRS, mean (SD)*	12.7 (8.9)	2.1 (2.8)	0.001
*ASSIST, mean (SD)*	29.7 (26.4)	17.9 (19.9)	0.006
*antipsychotic olanzapine equivalent dose, mg, mean (SD)*	19 (25.4)	–	–
*antidepressant fluoxetine equivalent dose, mg, mean (SD)*	13.3 (25)	–	–

*ASSIST* Alcohol, Smoking and Substance Involvement Screening Test, *BPRS* Brief Psychiatric Rating Scale, *MADRS* Montgomery–Åsberg Depression Rating Scale, *SANS* Scale for the Assessment of Negative Symptoms, *SD* standard deviation, *SOFAS* Social and Occupational Functioning Assessment Scale.

### ROI-based QSM comparison

**Fig. 1:**
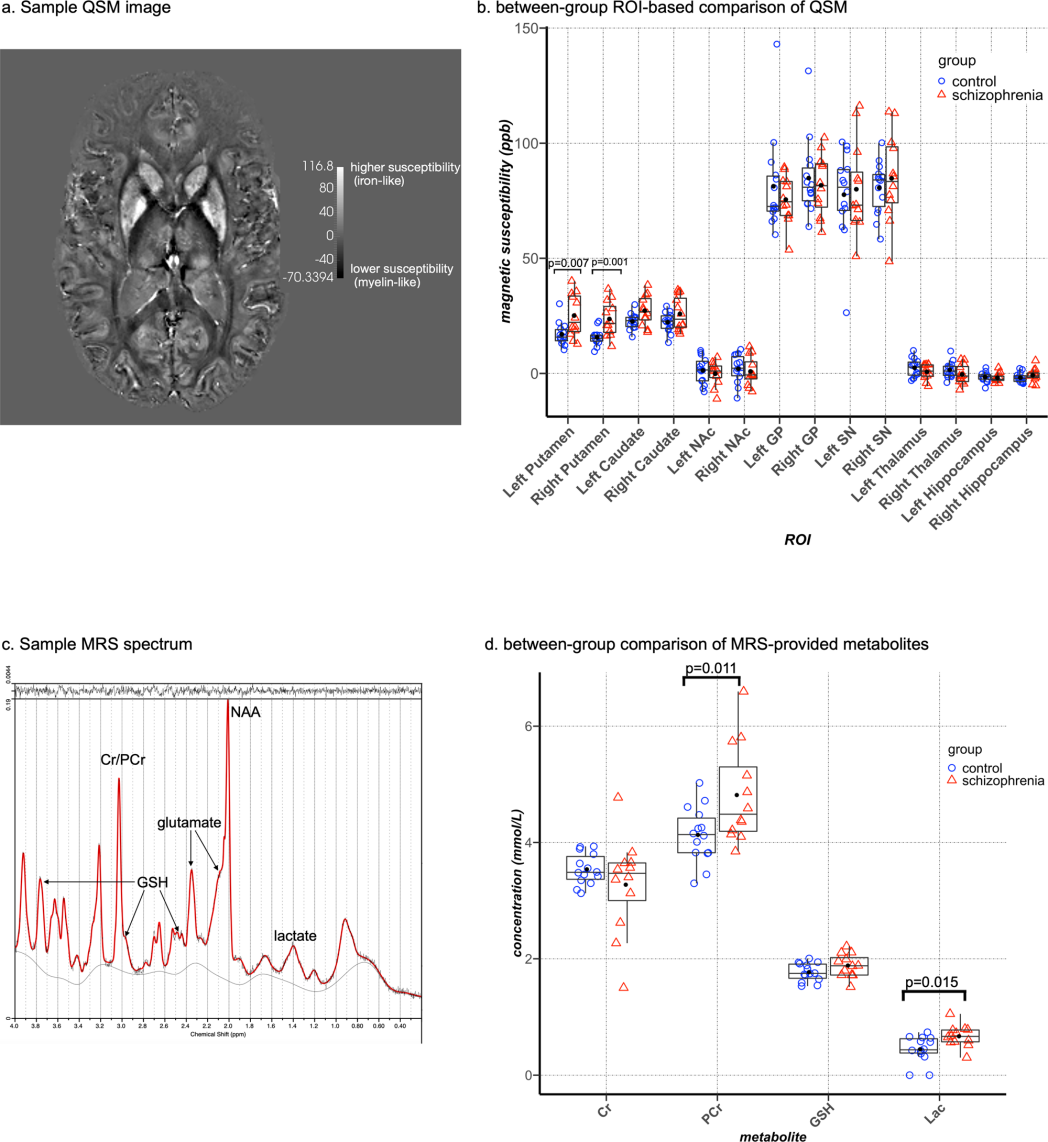
Between-group comparison of regional QSM and MRS metabolites. **a** a representative QSM image from our dataset. Regions with higher magnetic susceptibility (higher levels of iron) appear brighter. **b** Tukey’s boxplots overlaid on dot plots of ROI-based comparison of mean susceptibility values between healthy controls (blue circles) and individuals with schizophrenia (red triangles). Black dots represent the mean values in each group. High signal intensity in the SN and GP indicates high iron content in these regions in both groups. **c** a representative MRS spectrum from our dataset (**d**). Tukey’s boxplots overlaid on dot plots demonstrating the between-group comparison of neurometabolites in the daCC. Blue circles represent healthy controls and red triangles represent individuals with schizophrenia. Black dots indicate mean values in each group. SN substantia nigra, GP globus pallidus, NAc nucleus accumbens. Cr creatine, PCr phosphocreatine, GSH glutathione, ppb parts per billion.

We compared mean QSM values in the putamen, caudate nucleus, globus pallidus, thalamus, hippocampus, substantia nigra, and nucleus accumbens between groups as our ROI-based comparison. Figure 1a shows a representative QSM construction from our dataset. After correction for multiple comparisons, significantly higher magnetic susceptibility was observed in the putamen bilaterally with large effect size (left: *p* = 0.007, η^2^_p_ = 0.3; right: *p* = 0.001, η^2^_p_ = 0.42) in individuals with schizophrenia. QSM signal in the left caudate nucleus was also higher in schizophrenia (*p* = 0.023, η^2^_p_ = 0.223), however, this did not survive correction for multiple comparisons. Comparison of QSM values in the other ROIs did not reveal any significant differences (Table 2 and Fig. 1b).

**Table 2:** Comparison of mean regional QSM values (ppb) between groups.

ROI			control (*n* = 14)	schizophrenia (*n* = 12)	df	F	*p* value	effect size η^2^_p_	Observed power
putamen	left	mean (SD)	17 (5)	25.1 (9.3)	**(1, 21)**	**9.01**	**0.007**	**0.3**	**0.89**
	right	mean (SD)	15.6 (3.5)	23.6 (7.6)	**(1, 21)**	**15.2**	**0.001**	**0.42**	**0.98**
caudate nucleus	left	mean (SD)	22.6 (3.4)	27.2 (6.5)	(1, 21)	6.02	0.02	0.22	0.73
	right	mean (SD)	22.2 (4.3)	25.8 (7.3)	(1, 21)	2.3	0.14	0.09	0.33
nucleus accumbens	left	mean (SD)	1.3 (5.9)	−0.1 (5.1)	(1, 21)	0.1	0.7	−0.007	
	right	mean (SD)	2 (6.1)	0.9 (6.3)	(1, 21)	0.1	0.75	−0.005	
globus pallidus	left	mean (SD)	81.3 (20.8)	75.5 (10.4)	(1, 21)	0.3	0.26	−0.06	
	right	mean (SD)	84.8 (16.6)	81.7 (13.2)	(1, 21)	0.4	0.53	−0.02	
substantia nigra	left	mean (SD)	77.9 (17.2)	76.5 (9.9)	(1, 21)	1.9	0.21	−0.08	
	right	mean (SD)	79 (13.2)	82.6 (10.7)	(1, 21)	0.02	0.82	0.001	
thalamus	left	mean (SD)	2.4 (3.9)	0.7 (3.3)	(1, 21)	0.07	0.79	−0.003	
	right	mean (SD)	1.5 (3.5)	−0.4 (4.2)	(1, 21)	1.2	0.28	−0.05	
hippocampus	left	mean (SD)	−1.5 (2.1)	−2 (2)	(1, 21)	0.01	0.91	−0.001	
	right	mean (SD)	−2 (2)	−0.8 (2.9)	(1, 21)	1.7	0.21	0.07	

*df* degrees of freedom, *ppb* parts per billion, *SD* standard deviation. Statistical indices for significant between-group differences after adjustment for multiple comparisons are bolded.

### Post hoc voxel-wise QSM comparisons

The whole-brain voxel-wise comparisons of QSM between groups revealed a cluster of higher susceptibility in the right putamen (cluster size = 201 voxels, Montreal Neurological Institute (MNI) location x = 28.2 y = −3.53 z = 5.61) and a large cluster of lower susceptibility in the left lateral parts of the body of the corpus callosum (cluster size = 1203 voxels, MNI location x = −13.3 mm y = 13.2 mm z = 23.5 mm) in the schizophrenia group (Supplementary Fig. 2).

### Comparison of metabolites in the caCC between groups

Figure 1c shows a sample MRS spectrum from our dataset. Concentrations of phosphocreatine and lactate were both significantly higher in the schizophrenia group, consistent with increased anaerobic metabolism (phosphocreatine: Hedges’ g = 0.989, *p* = 0.023; lactate: Hedges’ g = 1.041, *p* = 0.012) (Table 3 and Fig. 1d). Comparison of GSH concentration, as a marker of oxidative stress, did not reveal any significant between-group differences.

**Table 3:** Comparison of metabolite concentrations (mmol/lit) between groups.

	control	schizophrenia	df	t	*p* value	Hedges’ g	Observed power
*metabolite*							
*PCr, mmol/L, mean (SD)*	4.1 (0.5)	4.8 (0.8)	**16.8**	**−2.5**	**0.02**	**0.99**	**0.68**
*Cr, mmol/L, mean (SD)*	3.5 (0.3)	3.3 (0.8)	12.9	1.06	0.31	0.43	
*GSH, mmol/L, mean (SD)*	1.8 (0.1)	1.9 (0.2)	24	−1.6	0.11	0.62	
*lactate, mmol/L, mean (SD)*	0.4 (0.2)	0.7 (0.2)	**24**	**−2.7**	**0.01**	**1.04**	**0.75**
*quality measures:*							
*linewidth (FWHM Hz), mean (SD)*	9.4 (2.1)	10.4 (1.8)			0.2	0.5	
*SNR, mean (SD)*	41.1 (13.2)	35.7 (9.5)			0.26	0.44	
*PCr CRLB, mmol/L, mean (SD)*	0.30 (0.08)	0.33 (0.08)			–	–	
*Cr CRLB, mmol/L, mean (SD)*	0.28 (0.08)	0.30 (0.07)			–	–	
*GSH CRLB, mmol/L, mean (SD)*	0.08 (0.02)	0.08 (0.02)			–	–	
*lactate CRLB, mmol/L, mean (SD)*	0.09 (0.05)	0.14 (0.04)			–	–	

*Cr* creatine, *CRLB* Cramer-Rao lower bound, *df* degrees of freedom, *GSH* glutathione, *FWHM* full width at half maximum, *PCr* phosphocreatine, *SD* standard deviation, *SNR* signal to noise ratio. Statistical indices for significant between-group differences after adjustment for multiple comparisons are bolded.

### Correlations between mean magnetic susceptibility of the regions within the associative and limbic striatal pathways

**Fig. 2:**
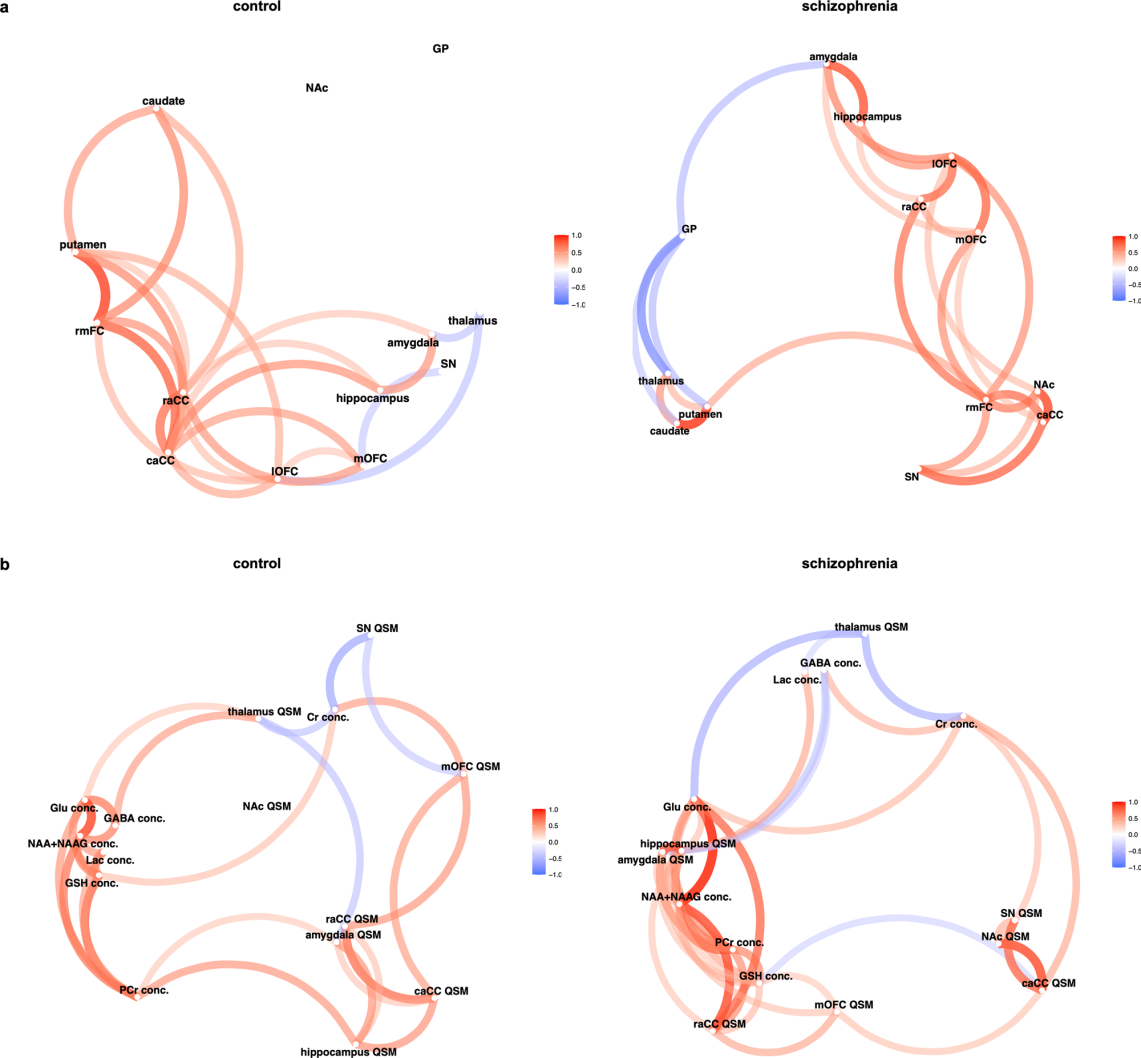
Correlation network plots for regional QSM and MRS measures in schizophrenia and control groups. **a** correlation network of QSM in the main regions of cortico-subcortical networks in the control (left) and schizophrenia group (right) – (**b**). correlation network of neurometabolites in the caCC and QSM in the regions related to the caCC in the control (left) and schizophrenia group (right). Only the correlations with Pearson coefficients higher than 0.4 are shown. caCC caudal anterior cingulate cortex, conc. concentration, Cr creatine, GABA gamma-aminobutyric acid, Glu glutamate, GP globus pallidus, GSH glutathione, Lac lactate, lOFC lateral orbitofrontal cortex, mOFC medial orbitofrontal cortex, NAA N-acetyl-aspartate, NAAG N-acetyl-aspartyl-glutamate, NAc nucleus accumbens, PCr phosphocreatine, raCC rostral anterior cingulate cortex, rmFC rostral middle frontal cortex, SN substantia nigra.

Our network plot visualization (Fig. 2a) of the correlations among QSM values in cortico-subcortical regions demonstrated that in healthy controls, the nucleus accumbens and globus pallidus were distanced (indicating low correlations) from the other regions, while the putamen and caudate nucleus had closer associations with the cortical ROIs. In schizophrenia, however, the nucleus accumbens showed strong correlations with the cortex, hippocampus, and amygdala, while the dorsal striatum, thalamus and globus pallidus were clustered together, farther away (weaker correlations) from the cortical ROIs.

We provide correlation matrices for each pair of the above correlations with *r* values, unadjusted and multiple comparison-adjusted p-values in Supplementary Fig. 3. After false detection rate (FDR) adjustment, in the schizophrenia group, among the ROIs related to the associative striatum, there were significant correlations between QSM values in the putamen and caudate nucleus (*r* = 0.88, *p* < 0.001). In the ROIs associated with the limbic striatum, mean magnetic susceptibility in the caCC correlated with QSM in nucleus accumbens (*r* = 0.83, *p* = 0.001) and substantia nigra (*r* = 0.75, *p* = 0.005). Further, there was a correlation between QSM values in the hippocampus and amygdala (*r* = 0.81, *p* = 0.001). We also found a significant negative correlation between QSM in the globus pallidus and thalamus, which are the common ROIs in the associative and limbic cortico-striato-thalamo-cortical (CSTC) pathways. In the control group, QSM values were significantly correlated only between the rostral middle frontal cortex (rmFC) and putamen (*r* = 0.85, *p* < 0.001) after adjustment for multiple comparisons. The statistical comparison of the correlation coefficients between groups did not show any significant differences.

### Correlations between QSM and clinical assessments and medications

After correction for multiple comparisons, there were no significant correlations between QSM and clinical assessments, antipsychotic, and antidepressant medications.

### Correlation between MRS and QSM

A qualitative representation of the patterns of correlations between MRS and QSM are provided in the network plots in Fig. 2b. While in healthy controls neurometabolites are clustered separately from QSM variables, in schizophrenia, the network plot shows clustering of QSM in the hippocampus, amygdala, and rostral anterior cingulate cortex (raCC) together with the neurometabolite concentrations. The correlation matrices with correlation coefficients and unadjusted p-values are provided in Supplementary Fig. 4. Significant p-values in this exploratory analysis did not survive correction for multiple comparisons.

### Regional correlation between QSM and ROI volume

**Fig. 3:**
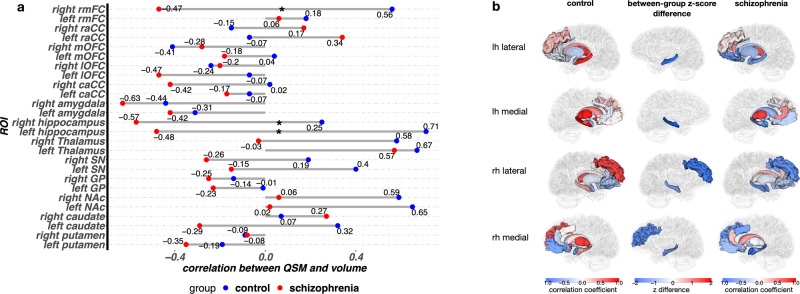
Correlation between mean QSM value and volume/thickness in ROIs within schizophrenia and control groups. **a** correlation between mean QSM and volume or thickness within each ROI in the control (left) and schizophrenia group (right). The middle column shows the difference of z scores after r to z transformation in the regions where the correlation between QSM and volume where significantly different between groups. (z difference) = (z schizophrenia) - (z control) (**b**). bar plot showing the correlation between QSM and volume/thickness in ROIs. Numbers indicate correlation coefficients and significant between-group differences are marked by asterisks. caCC caudal anterior cingulate cortex, GP globus pallidus, lOFC lateral orbitofrontal cortex, lh left hemisphere, mOFC medial orbitofrontal cortex, NAc nucleus accumbens, raCC rostral anterior cingulate cortex, rh right hemisphere, rmFC rostral middle frontal cortex, SN substantia nigra.

The results of our exploratory correlation analysis between mean magnetic susceptibility and volume or cortical thickness within each of the key cortical and subcortical ROIs for the schizophrenia and control groups is shown in Fig. 3. We observed between-group differences of these correlations in the right (r_control_ = 0.25, r_schizophrenia_ = −0.57, unadjusted *p* = 0.044) and left (r_control_ = 0.71, r_schizophrenia_ = −0.48, unadjusted *p* = 0.001) hippocampus, and right rmFC (r_control_ = 0.55, r_schizophrenia_ = −0.47, unadjusted *p* = 0.010). In all three of these ROIs, the correlation between QSM and volume/thickness was negative in schizophrenia (implying that iron was a burden) and positive (implying that iron was an asset) in controls. Statistical significance of these differences did not survive correction for multiple comparisons.

### Correlation between MRS and cortical thickness

In the control group, there were no observed correlations between MRS-derived metabolites and cortical gray matter thickness in any of the regions. In the schizophrenia group, however, lower GSH was associated with lower thickness in the raCC (*r* = 0.71, unadjusted *p* = 0.01). This significance did not survive correction for multiple comparisons (Supplementary Fig. 5).

## Discussion

To the best of our knowledge, this is the first in vivo study to investigate MRI indices of brain iron and markers of oxidative and metabolic stress in chronic schizophrenia. We primarily examined tissue iron content in subcortical gray matter regions where QSM signal shows strong correlation with tissue iron concentration, providing a reliable estimate of iron^21,28–31^. As expected, high QSM signal was identified in the substantia nigra (an iron-rich nucleus) both in healthy and schizophrenia groups. Compared with healthy controls, individuals with schizophrenia had higher iron content in the putamen with large effect size (η^2^_p_ = 0.3 and 0.42 for left and right putamen, respectively), as well as a trend for such an increase in the left caudate nucleus. Based on the identified regulatory role of iron in dopamine production^10^ and intracellular trafficking of dopamine receptor^13^, along with induction of cellular iron uptake by dopamine^12^, our finding is in line with our hypothesis that iron content would be increased in the dorsal striatum in individuals with schizophrenia^23^. A recent QSM study of individuals with first-episode medication-naïve schizophrenia detected lower iron in bilateral substantia nigra, left red nucleus, and thalamus^32^. The contrast between our findings and the study by Xu et al. can be due to the difference in the illness chronicity. Therefore, it warrants further studies using longitudinal QSM to observe the trajectory of iron changes in the course of schizophrenia.

**Fig. 4:**
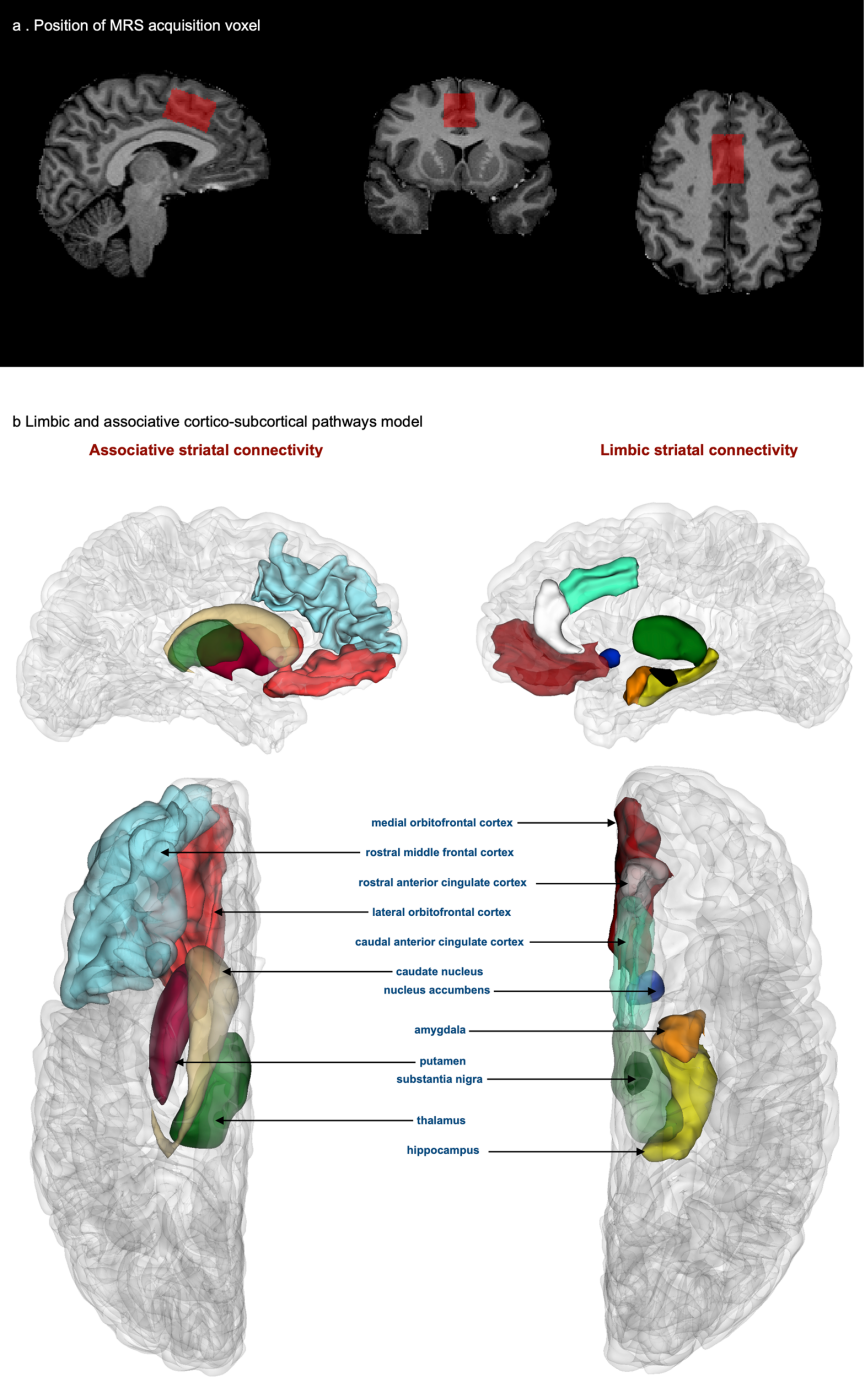
Neuroanatomical representation of MRS acquisition voxel and the ROIs comprising CSTC circuits. **a** location of the MRS acquisition voxel in the caCC (**b**). CSTC pathways that involve the limbic striatum (right) and associative striatum (left), top: medial view, bottom: superior view.

Despite the abundance of evidence on the role of iron in a range of functions in the CNS, the mechanisms for its regulation within the brain are not yet identified. A recent study identified an axonal iron trafficking mechanism in the mouse brain, which transports iron through neural fibers. The transport of iron through these pathways was shown to be associated with neuronal activity, medications, and anxiety-related behaviors, suggesting a dynamic and functional regulation of iron within the brain^33^. In light of this finding and considering the significance of the limbic and associative CSTC circuits (Fig. 4b) in schizophrenia, we conducted network-based structural covariance analysis of QSM within these circuits. The results of this analysis demonstrated different patterns of correlations in schizophrenia and control groups suggesting a network-wide effect on distribution of iron in the brains of patients with schizophrenia. We observed strong correlations between the cortex and striatum in the associative CSTC in healthy controls but not in schizophrenia, where this correlation was stronger in the limbic CSTC (Fig. 2a and Supplementary Fig. 3). We speculate that the absence of correlations between cortex and striatum in the associative CSTC in schizophrenia could be related to the disrupted functional connectivity that has been reported between these two regions in previous studies^34^, especially if neural activity-dependent iron trafficking pathways exist in the human brain. Our findings warrant further explorations in larger studies implementing functional MRI and QSM to provide better understanding of the dynamics of iron regulation in association with neuronal activity in physiologic and pathologic conditions.

Excess iron can generate oxidative stress and lead to mitochondrial dysfunction. To examine these metabolic abnormalities, we used MRS to measure the concentrations of GSH and lactate as markers of oxidative stress and anaerobic metabolism (an indicator of mitochondrial dysfunction), respectively. In the MRS acquisition voxel placed within the caCC, the concentration of GSH did not differ between groups, but lactate was increased in schizophrenia suggesting an energy deficit state and elevated anaerobic metabolism^35^. Existing in vivo evidence on GSH alterations in schizophrenia is inconsistent. While some studies have reported lower GSH levels consistent with increased oxidative stress in schizophrenia, others have not found a difference^36^. The brain region where MRS acquisition is performed appears to contribute to the inconsistencies in the literature. Our finding of increased lactate is in line with a previous ultra-high field MRS study that reported increased lactate in the anterior cingulate cortex in schizophrenia^37^. One of the cellular abnormalities that can lead to elevated anaerobic metabolism is defective oxidative phosphorylation arising from mitochondrial dysfunction. Abnormal number and function of mitochondria as well as mitochondrial fragmentation have been identified in schizophrenia^38,39^. While excess iron has been shown to be associated with mitochondrial dysfunction^40^, our study was not able to directly examine the link between iron accumulation and anaerobic metabolism since these two findings were observed in separate brain regions. However, we conducted a network correlation model between neurometabolites in the caCC and QSM in the regions that are functionally or structurally connected to the caCC. In this model, schizophrenia patients showed stronger correlations between the hippocampus, amygdala, and raCC with the neurometabolites in the caCC, while healthy controls demonstrated a segregation of QSM and neurometabolite concentrations. This finding suggests that a network-wide dysregulation of iron from its physiologic equilibrium can potentially be associated with alterations in neurometabolic status. Future studies with larger sample sizes using QSM and MRS can provide a clearer picture on the potential association between iron disturbances and metabolic imbalance.

Although the focus of this study was to primarily evaluate brain iron in the gray matter using QSM, post hoc voxel-wise analysis revealed widespread changes of magnetic susceptibility in the lateral aspects of the corpus callosum. In the white matter, iron is not the sole determinant of magnetic susceptibility, and alterations in QSM value can be induced by changes in both myelin and iron, with diamagnetic myelin contributing negatively to total magnetic susceptibility. In heavily myelinated fibers, alterations in myelination, microstructure, and the orientation of white matter tracts in relation to the external magnetic field affect the apparent magnetic susceptibility^41^. Hence, pathological mechanisms resulting in white matter microstructural changes, such as white matter fiber thinning, free water alterations, and myelin disintegration can have unpredictable effects on the estimated susceptibility. Diffusion-weighted imaging (DWI) has provided evidence suggesting myelin abnormalities, reduced fiber integrity^42,43^, and reduced fiber compactness in the corpus callosum in schizophrenia^44^, all of which may have given rise to our finding of lower QSM in the corpus callosum. This finding suggests that studies investigating white matter changes in schizophrenia are likely to benefit from the information provided by magnetic susceptibility changes in addition to DWI measures for a clearer picture of white matter pathologies of schizophrenia.

This study had several limitations. First, our small study population has limited statistical power and only the highest effect sizes reached statistical significance between groups. A larger sample size may have been able to reveal smaller between-group differences in other regions, especially in the caudate nucleus where we found a trend for increased iron levels. In the analysis of correlations among brain regions within the striatal pathways, the small sample size prevented the evaluation of partial correlations, as that statistical approach demands that the number of ROIs is smaller than the sample size. Another limitation was the lack of cognitive assessments. Since the associative striatum is central to cognitive functions, such information would have enabled examining the correlation between iron in the dorsal striatal circuits and cognitive deficits. Finally, in this study, we acquired MRS in a voxel within the caCC, which has shown reliable MRS signal at 7-Tesla in our previous studies^45,46^. QSM, on the other hand, shows the highest accuracy for tissue iron in the subcortical gray matter, and correlates less strongly with tissue iron in the cortical gray matter. Therefore, we were not able to examine whether the large group difference in iron content within the putamen is locally associated with metabolic changes. However, we conducted correlation analyses between QSM, neurometabolite concentrations, and ROI volume/thickness as exploratory tests to inform and generate hypotheses for the future studies.

In conclusion, our study provides evidence of elevated iron levels in the dorsal striatum, associated with a network-wide impact on iron distribution within other brain regions. This is likely to occur in association with abnormal dopaminergic activity and possibly antipsychotic medications. Iron accumulation can generate oxidative stress and lead to mitochondrial dysfunction and neuronal loss. Multimodal MRI studies using QSM and MRS within the striatum can further inform whether this excess iron is associated with pathologic metabolic changes.

## Methods

### Study population

This study was approved by Melbourne Health Human Research Ethics Committee (HREC) (Project 2012.066) and Australian Research Infrastructure network (ARIN 7T-2015.005, CFMS No. MRI495000045). From September of 2016 to December of 2019, 12 individuals with chronic schizophrenia from Northwestern Mental Health services and 14 healthy individuals with similar age and sex were recruited as the control group in the study. Written informed consent was obtained from all participants prior to enrollment.

Inclusion criteria for the schizophrenia group were: more than five years of illness following an initial DSM-IV diagnosis of schizophrenia; age between 18–50 years; stabilized on antipsychotic treatment for at least six weeks. Exclusion criteria for all subjects were: history of significant head injury; neurological disease, including seizures; impaired thyroid function; diabetes; premorbid IQ < 70, developmental delay, or intellectual disability; systemic inflammatory conditions; pregnancy, breastfeeding; and MRI-related contraindications, such as, magnetic metal implants and claustrophobia. Additional exclusion criteria for healthy controls were personal or family (first-degree relative) history of a psychiatric or neurological illness, significant psychopathology, and past or current use of any psychoactive medications.

### Clinical evaluations

A trained clinician performed psychological batteries including the Brief Psychiatric Rating Scales (BPRS) for evaluation of a range of psychiatric symptoms, the Montgomery-Åsberg Depression Rating Scale (MADRS) for assessment of depressive symptoms, the Scale for the Assessment of Negative Symptoms (SANS), the Alcohol Smoking and Substance Involvement Screening Test (ASSIST), and the Social and Occupational Functioning Assessment Scale (SOFAS). These measures were collected in both groups. A venous blood sample was also obtained for measurement of serum iron levels on the same day as the brain MRI acquisition and clinical psychological assessments.

### Serum iron quantification

Fifty microliters of serum were lyophilized for 12 h and were digested using nitric acid under heat conditions. Hydrogen peroxide was then added to each sample, and they were heated again to 90 °C. Measurements were made using an Agilent 7700 series Inductively Coupled Plasma-Mass Spectrometry (ICP-MS) instrument under routine multi-element operating conditions, using a helium reaction gas cell. For each sample, iron levels were expressed in μmol/L, which is consisted of all components of iron in the serum, including free and protein-bound forms.

### Imaging methods

MRI acquisitions were performed using a 7-Tesla MRI scanner (Magnetom 7 T, Siemens Healthcare, Erlangen, Germany) with a 1Tx/32Rx head coil (Nova Medical Inc.). No software or hardware updates or modifications were made to the MRI scanner throughout the data acquisition for this study.

For the T_1_ MRI, a Magnetization Prepared Two Rapid Acquisition Gradient (MP2RAGE) sequence was obtained with the following parameters: echo time (TE) = 2.89 ms, repetition time (TR) = 4900 ms, matrix size = 256 × 232 × 192, voxel size = 0.9 × 0.9 × 0.9 mm, acquisition time = 5:25 min. For QSM construction, a multiecho Gradient Echo (GRE) sequence was acquired with the following parameters: number of echoes = 9, first TE = 5.1 ms, DTE = 2.04 ms, TR = 24 ms, flip angle = 13°, matrix size = 280 × 242 × 192, voxel size = 0.75 mm isotropic, acquisition time = 8:42 min.

Single voxel ^1^H MRS was acquired using the STimulated Echo Acquisition Mode (STEAM) method with (32 averages) and without (4 averages) water suppression, with the following parameters: TE = 6 ms, TR = 8500 ms, mixing time (TM) = 32 ms, 1,024 points, 6 kHZ and Variable Power RF Pulses With Optimized Relaxation Delays (VAPOR)^47^ water suppression. MRS was obtained from a midline 20 × 30 × 20 mm voxel placed within the caudal anterior cingulate cortex (caCC) in the midsagittal slice. The caCC is one of the key cortical regions implicated in schizophrenia associated with limbic corticostriatal circuitry, and provides robust MRS acquisitions^48,49^. The longest dimension of the MRS voxel was aligned above and parallel to the superior border of the body of the corpus callosum and the anterior border of the voxel was leveled with the posterior of the genu of the corpus callosum (Fig. 4a).

### Image processing

#### Segmentation

T_1_ images were used for anatomical labeling and segmentation of the regions of interest (ROIs). Skull stripping was performed using the multi-atlas brain segmentation (MABS) brain masking tool^50^ (https://github.com/pnlbwh/PNL-manual#multi-atlas-brain-segmentation-mabs), followed by visual quality control and manual correction using ITK-SNAP v3.8 (http://www.itksnap.org/)^51^ by an investigator blind to subjects’ group membership (patient/control).

We performed automatic brain segmentation followed by manual correction. Subcortical and hippocampal segmentation was performed using the Multiple Automatically Generated Templates Brain Segmentation pipeline (MAGeT-brain v1.0 https://github.com/CoBrALab/MAGeTbrain)^52^. MAGeT-brain can employ multiple atlases for segmentation. We used the 5-brain atlas provided with the MAGeT-brain pipeline for thalamic and hippocampal segmentation^53,54^ and the CIT168 atlas^55^ for the striatum (nucleus accumbens, caudate, putamen), substantia nigra, and globus pallidus. Automatic segmentation of the cortex and amygdala was performed using FreeSurfer v7.1.0^56^.

The outputs of automatic segmentation tools were visually inspected. To ensure the accuracy of the parcellations, label maps were manually corrected in the striatum, globus pallidus, thalamus, hippocampus, and amygdala using their morphologic appearance in T_1_-weighted images. Due to the high signal contrast of the substantia nigra in QSM, we used QSM images to guide manual tracing of the substantia nigra. Manual editing was performed using 3D Slicer v4.11 (https://www.slicer.org)^57^ by an investigator (P.R.) under guidance and quality assessment of two senior neuroanatomists (N.M. and R.J.R.), all of whom were blinded to group membership of subjects and following neuroanatomical criteria reported in previous publications of our group^58–60^. The temporal pole, fusiform, inferior temporal, and entorhinal cortices were excluded from further analyses due to unreliable parcellation.

#### Quantitative Susceptibility Mapping (QSM) processing

QSM estimation was performed from the magnitude and phase images of the multi-echo GRE sequence using the Quantitative Susceptibility Mapping Artifact Reduction Technique (QSMART https://github.com/wtsyeda/QSMART)^61^. A detailed description of QSMART can be found elsewhere^61^. Briefly, a brain mask is generated using FSL’s Brain Extraction Tool (FMRIB, Oxford University, UK)^62^. Phase data is unwrapped using a Laplacian-based method^63^, background field is removed using 3D spatially dependent filtering^64^, followed by a magnitude-weighted least squares method for dipole inversion (iLSQR)^65^. For each subject, a rigid transform from the non-skull-stripped T_1_ image to the first echo magnitude image of the GRE acquisition was created using Advanced Normalization Tools (ANTs) v.2.3.5 (http://stnava.github.io/ANTs/)^66^ and applied to the label maps to align segmentations onto the QSM images. Mean intensity values within each ROI were extracted and used for further analyses.

#### Magnetic Resonance Spectroscopy (MRS) processing

We used LCModel version 6.3-1 M (http://s-provencher.com/lcmodel.shtml)^67^, using a batch script from OpenMRSLab (https://github.com/openmrslab), with a 7-T basis set created based on the scanner and sequence parameters provided by the LCModel support team, and validated in a previous study^45^. Scaling to unsuppressed water and Eddy current correction were performed. Using the FreeSurfer outputs from the brain segmentation step, tissue composition of the MRS voxel was determined and CSF partial volume correction of the metabolite concentrations was performed as described by S. Provencher, (2009)^68^. Quality assessments of the MRS spectra were performed by a senior investigator (A.P.L.) blinded to participants’ diagnosis to identify any major spectral artifacts. A threshold of SNR greater than 10 and linewidth lower than 30 Hz were considered as minimum satisfactory quality of the MRS acquisitions and all spectra surpassed this threshold. In addition, the reliability of each metabolite measurement was assessed by assessing Cramer-Rao lower bound (CRLB) as a function of the concentration. Due to the potential to bias data, data points were not removed but CRLBs are reported in Table 3^69^. Concentrations of phosphocreatine, creatine, GSH, and lactate were used as the primary measures for comparison between groups. For the exploratory evaluation of the correlation between MRS, QSM and volume, we also included concentrations of gamma-aminobutyric acid (GABA), glutamate, and N-acetyl aspartate (NAA) in the analyses.

### Statistical analysis

Participants’ characteristics such as age, serum iron, body mass index (BMI), and clinical assessments were compared between groups using two-tailed independent samples t-test for continuous variables and Chi-square test for sex. ROI volumes were compared between groups using univariate analysis of covariance (ANCOVA) with total brain volume, obtained by the summation of gray and white matter volumes, as a covariate.

We compared regional mean QSM values in the putamen, caudate nucleus, globus pallidus, thalamus, hippocampus, substantia nigra, and nucleus accumbens among groups using ANCOVA with age, serum iron and ROI volume as covariates. Serum iron was included in the statistical model to account for the QSM contribution of the circulating serum iron within the ROIs. Between-group effect sizes of differences were calculated and reported as partial eta square (η^2^_p_). We evaluated the correlation between mean QSM values in these ROIs and clinical assessments using the Pearson test. These analyses were performed in SPSS (version 27; IBM SPSS Statistics for Mac, IBM Corp., Armonk, NY, USA).

For a post-hoc whole-brain voxel-wise comparison of QSM between healthy and schizophrenia groups, a study template was created from the T_1_ images of all participants using ANTs (Avants & Gee, 2004). For each subject, a rigid transform was applied to reposition the QSM image to the native T_1_ space, followed by an affine+nonlinear transformation to the common study template. All images were moved to the MNI 152 space. To identify voxel clusters of significant between-group differences in QSM images, FSL’s randomise tool was used with 5,000 permutations and family-wise error rate (FWER) correction with TFCE clustering^62,70,71^.

From the MRS acquisition, we compared the concentrations of creatine, phosphocreatine, lactate, and GSH between groups using two-tailed independent t-tests in SPSS version 27 and effect sizes of differences were reported as Hedges’ g.

We used a structural covariance analysis to examine the patterns of correlations among mean QSM values of ROIs within the cortico-subcortical pathways of limbic and associative striatum. In the model, we included regions that are associated with the striatum based on the CSTC circuits^72^ implicated in schizophrenia (see Dandash et al., 2017). The limbic striatum pathways model consisted of the nucleus accumbens, thalamus, substantia nigra, insula, mOFC, caCC, raCC, hippocampus, and amygdala. Our model of the associative pathways included the putamen, caudate nucleus, thalamus, substantia nigra, lateral orbitofrontal cortex (lOFC), and rmFC (Fig. 4b). To increase the statistical power in this analysis, we used mean QSM values extracted from the combined right and left side ROI labels. Pearson correlation tests were conducted. Fisher’s r to z transformations were performed and tested for equality using t-tests to compare the pair-wise ROI correlations between groups. All of the above statistical tests were considered primary analyses and correction for multiple comparisons was performed using the Benjamini and Hochberg^73^ method with an FDR of 0.05. For a qualitative visualization of the patterns of correlations among variables, we used network plots that use a multidimensional clustering method^74^. Data processing, analysis and visualizations for this step was performed in RStudio 2021.09.0 Build 351 “Ghost Orchid” Release^75^ and R version 4.1.2, using the psych 2.1.9^76^, corrr 1.0.6.1^74^, corrplot 0.92^77^, corx 1.0.6.1^78^, tidyverse 1.3.1^79^, readxl 1.3.^80^, ggplot2^81^, and dplyr 1.0.7^82^ packages.

We further conducted exploratory Pearson correlation tests (without correction for multiple comparisons) using the above packages in RStudio, to evaluate the correlations among QSM signal, neurometabolite concentrations and volumetric measures. To examine the correlation between QSM and MRS, we performed correlation tests between neurometabolite concentrations (creatine, phosphocreatine, GSH, lactate, glutamate, GABA, and NAA + NAAG (concentrations of both NAA and N-Acetylaspartylglutamate (NAAG)) and mean QSM values in the cortical and subcortical structures associated with the anterior cingulate cortex (raCC, caCC, mOFC, hippocampus, amygdala, nucleus accumbens, thalamus and substantia nigra). To evaluate the association between QSM and volumetric changes, we tested the correlation between mean QSM value and cortical thickness or subcortical volume within each of the cortical and subcortical ROIs that constitute our cortico-subcortical striatal connectivity model shown in Fig. 4b. Finally, to explore the association between MRS and cortical thickness, we performed correlation tests between the concentrations of neurometabolites (creatine, phosphocreatine, GSH, lactate, glutamate, GABA, and N-acetyl aspartate (NAA + NAAG)) and the weighted average of right and left hemisphere cortical thickness in the raCC, caCC, and superior frontal areas, that comprised the MRS voxel.

### Post hoc power analysis

To determine the achieved power of the primary comparative statistical analyses, we used G*Power software Version 3.1.9.6. For between-group ANCOVA QSM comparisons, we used the following input parameters: α = 0.05, numerator df = 1, total sample size = 26, number of groups = 2, number of covariates = 3, and effect size (η_p_^2^) in each ROI. With a power of 0.8, the minimum detectable effect size of f was 0.58, critical F = 4.32, and noncentrality parameter λ = 8.63. The following parameters were used for t-test MRS comparisons: group 1 size = 12, group 2 size = 14, α = 0.05, and effect size (d) in each ROI. With a power of 0.8, the minimum detectable effect size of d was 1.14, critical t = 2.06, and noncentrality parameter δ = 2.92.

## Supplementary information

Suplemental Material

## Data Availability

The datasets that support the findings of this study consist of individuals’ neuroimaging data, which are not publicly available per the conditions of the Human Research Ethics Committee approval. Anonymized neuroimaging data and extracted metrics generated from processed data, however, are available from the authors upon reasonable request.

## Code availability

All software packages used in this study for data processing are publicly available and can be accessed via the URLs provided in the main text.

## References

[1] Bitanihirwe BKY, Woo T-UW (2011). Oxidative Stress in Schizophrenia: An Integrated Approach. Neurosci Biobehav Rev.

[2] Marques TR (2019). Neuroinflammation in schizophrenia: meta-analysis of in vivo microglial imaging studies. Psychol Med.

[3] Dwir D (2020). MMP9/RAGE pathway overactivation mediates redox dysregulation and neuroinflammation, leading to inhibitory/excitatory imbalance: a reverse translation study in schizophrenia patients. Mol Psychiatry.

[4] Rajasekaran A, Venkatasubramanian G, Berk M, Debnath M (2015). Mitochondrial dysfunction in schizophrenia: pathways, mechanisms and implications. Neurosci Biobehav Rev.

[5] Hare D, Ayton S, Bush A, Lei P (2013). A delicate balance: Iron metabolism and diseases of the brain. Frontiers in Aging Neuroscience.

[6] Hare DJ, Double KL (2016). Iron and dopamine: a toxic couple. Brain.

[7] Mairuae N, Connor JR, Cheepsunthorn P (2011). Increased cellular iron levels affect matrix metalloproteinase expression and phagocytosis in activated microglia. Neurosci Lett.

[8] Ward RJ, Dexter DT, Crichton RR (2022). Iron, Neuroinflammation and Neurodegeneration. Int J Mol Sci.

[9] Howes OD, Kapur S (2009). The dopamine hypothesis of schizophrenia: version III–the final common pathway. Schizophr Bull.

[10] Frantom PA, Seravalli J, Ragsdale SW, Fitzpatrick PF (2006). Reduction and oxidation of the active site iron in tyrosine hydroxylase: kinetics and specificity. Biochemistry.

[11] Kaushik P, Gorin F, Vali S (2007). Dynamics of tyrosine hydroxylase mediated regulation of dopamine synthesis. J Comput Neurosci.

[12] Dichtl S (2018). Dopamine promotes cellular iron accumulation and oxidative stress responses in macrophages. Biochemical Pharmacology.

[13] Unger EL, Wiesinger JA, Hao L, Beard JL (2008). Dopamine D2 Receptor Expression Is Altered by Changes in Cellular Iron Levels in PC12 Cells and Rat Brain Tissue. The Journal of Nutrition.

[14] Ward RJ, Zucca FA, Duyn JH, Crichton RR, Zecca L (2014). The role of iron in brain ageing and neurodegenerative disorders. Lancet Neurol.

[15] Stockwell BR (2017). Ferroptosis: A Regulated Cell Death Nexus Linking Metabolism, Redox Biology, and Disease. Cell.

[16] Pantelis C (2005). Structural brain imaging evidence for multiple pathological processes at different stages of brain development in schizophrenia. Schizophr Bull.

[17] Shenton ME, Dickey CC, Frumin M, McCarley RW (2001). A review of MRI findings in schizophrenia. Schizophr Res.

[18] Casanova MF, Comparini SO, Kim RW, Kleinman JE (1992). Staining intensity of brain iron in patients with schizophrenia: A postmortem study. Journal of Neuropsychiatry and Clinical Neurosciences.

[19] Casanova MF, Waldman IN, Kleinman JE (1990). A postmortem quantitative study of iron in the globus pallidus of schizophrenic patients. Biological psychiatry.

[20] Lange, K. W. *et al*. Brain iron and schizophrenia. in 37–43 (Springer, Vienna, 1993). 10.1007/978-3-7091-9322-8_3.

[21] Hametner S (2018). The influence of brain iron and myelin on magnetic susceptibility and effective transverse relaxation - A biochemical and histological validation study. Neuroimage.

[22] Ravanfar, P. et al. Systematic Review; Quantitative Susceptibility Mapping (QSM) of Brain Iron Profile in Neurodegenerative Diseases. *Front. Neurosci*. **15**, 618435 (2021). 10.3389/fnins.2021.618435 PMC7930077 33679303

[23] Dandash O, Pantelis C, Fornito A (2017). Dopamine, fronto-striato-thalamic circuits and risk for psychosis. Schizophr Res.

[24] Sun D (2009). Progressive brain structural changes mapped as psychosis develops in ‘at risk’ individuals. Schizophr Res.

[25] Sun D (2009). Brain surface contraction mapped in first-episode schizophrenia: a longitudinal magnetic resonance imaging study. Mol Psychiatry.

[26] Heckers S, Konradi C (2002). Hippocampal neurons in schizophrenia. J Neural Transm.

[27] McHugo M (2019). Hyperactivity and Reduced Activation of Anterior Hippocampus in Early Psychosis. Am J Psychiatry.

[28] Langkammer C (2012). Quantitative susceptibility mapping (QSM) as a means to measure brain iron? A post mortem validation study. NeuroImage.

[29] Lee H, Baek S-Y, Chun SY, Lee J-H, Cho H (2018). Specific visualization of neuromelanin-iron complex and ferric iron in the human post-mortem substantia nigra using MR relaxometry at 7T. Neuroimage.

[30] Lewis MM (2018). Susceptibility MRI captures nigral pathology in patients with parkinsonian syndromes. Mov Disord.

[31] Sun H (2015). Validation of quantitative susceptibility mapping with Perls’ iron staining for subcortical gray matter. Neuroimage.

[32] Xu M (2021). Brain iron assessment in patients with First-episode schizophrenia using quantitative susceptibility mapping. NeuroImage: Clinical.

[33] Wang Z (2019). Axonal iron transport in the brain modulates anxiety-related behaviors. Nature Chemical Biology.

[34] Fornito A, Zalesky A, Pantelis C, Bullmore ET (2012). Schizophrenia, neuroimaging and connectomics. Neuroimage.

[35] Lunsing RJ, Strating K, de Koning TJ, Sijens PE (2017). Diagnostic value of MRS-quantified brain tissue lactate level in identifying children with mitochondrial disorders. Eur Radiol.

[36] Duarte JMN, Xin L (2019). Magnetic Resonance Spectroscopy in Schizophrenia: Evidence for Glutamatergic Dysfunction and Impaired Energy Metabolism. Neurochem Res.

[37] Rowland LM (2016). Elevated brain lactate in schizophrenia: a 7 T magnetic resonance spectroscopy study. Transl Psychiatry.

[38] Flippo KH, Strack S (2017). An emerging role for mitochondrial dynamics in schizophrenia. Schizophr Res.

[39] Roberts RC (2017). Postmortem studies on mitochondria in schizophrenia. Schizophr Res.

[40] Onukwufor JO, Dirksen RT, Wojtovich AP (2022). Iron Dysregulation in Mitochondrial Dysfunction and Alzheimer’s Disease. Antioxidants (Basel).

[41] Groeschel S (2016). Assessing White Matter Microstructure in Brain Regions with Different Myelin Architecture Using MRI. PLoS One.

[42] Oestreich LKL (2017). Characterizing white matter changes in chronic schizophrenia: A free-water imaging multi-site study. Schizophr Res.

[43] Whitford TJ (2010). Corpus callosum abnormalities and their association with psychotic symptoms in patients with schizophrenia. Biol Psychiatry.

[44] Knöchel C (2012). Interhemispheric hypoconnectivity in schizophrenia: fiber integrity and volume differences of the corpus callosum in patients and unaffected relatives. Neuroimage.

[45] Gonen OM (2020). Reproducibility of Glutamate, Glutathione, and GABA Measurements in vivo by Single-Voxel STEAM Magnetic Resonance Spectroscopy at 7-Tesla in Healthy Individuals. Frontiers in Neuroscience.

[46] Gonen OM (2020). Seven-tesla quantitative magnetic resonance spectroscopy of glutamate, γ-aminobutyric acid, and glutathione in the posterior cingulate cortex/precuneus in patients with epilepsy. Epilepsia.

[47] Tkác I, Starcuk Z, Choi IY, Gruetter R (1999). In vivo 1H NMR spectroscopy of rat brain at 1 ms echo time. Magn Reson Med.

[48] Lahti AC (2006). Correlations between rCBF and symptoms in two independent cohorts of drug-free patients with schizophrenia. Neuropsychopharmacology.

[49] Reid MA (2010). Assessments of function and biochemistry of the anterior cingulate cortex in schizophrenia. Biol Psychiatry.

[50] Billah, T., Rathi, Y. & Bouix, S. NIFTI MRI processing pipeline, https://github.com/pnlbwh/pnlNipype. (2019) 10.5281/zenodo.3258854.

[51] Yushkevich PA (2006). User-guided 3D active contour segmentation of anatomical structures: significantly improved efficiency and reliability. Neuroimage.

[52] Chakravarty MM (2013). Performing label-fusion-based segmentation using multiple automatically generated templates. Hum Brain Mapp.

[53] Pipitone J (2014). Multi-atlas segmentation of the whole hippocampus and subfields using multiple automatically generated templates. Neuroimage.

[54] Tullo S (2018). Warping an atlas derived from serial histology to 5 high-resolution MRIs. Sci Data.

[55] Pauli WM, Nili AN, Tyszka JM (2018). A high-resolution probabilistic in vivo atlas of human subcortical brain nuclei. Sci Data.

[56] Fischl B (2012). FreeSurfer. Neuroimage.

[57] Fedorov A (2012). 3D Slicer as an image computing platform for the Quantitative Imaging Network. Magn Reson Imaging.

[58] Ahn MS (2007). Anatomic brain magnetic resonance imaging of the basal ganglia in pediatric bipolar disorder. J Affect Disord.

[59] Fischl B (2002). Whole brain segmentation: automated labeling of neuroanatomical structures in the human brain. Neuron.

[60] Makris N (1999). MRI-Based topographic parcellation of human cerebral white matter and nuclei II. Rationale and applications with systematics of cerebral connectivity. Neuroimage.

[61] Yaghmaie N (2021). QSMART: Quantitative susceptibility mapping artifact reduction technique. NeuroImage.

[62] Jenkinson M, Beckmann CF, Behrens TEJ, Woolrich MW, Smith SM (2012). FSL. Neuroimage.

[63] Li W, Wu B, Liu C (2011). Quantitative susceptibility mapping of human brain reflects spatial variation in tissue composition. NeuroImage.

[64] Ng A (2011). Spatially dependent filtering for removing phase distortions at the cortical surface. Magnetic Resonance in Medicine.

[65] Li W (2015). A method for estimating and removing streaking artifacts in quantitative susceptibility mapping. Neuroimage.

[66] Avants B, Gee JC (2004). Geodesic estimation for large deformation anatomical shape averaging and interpolation. Neuroimage.

[67] Provencher SW (1993). Estimation of metabolite concentrations from localized in vivo proton NMR spectra. Magn Reson Med.

[68] Provencher, S. LCModel and LCMgui User’s Manual. (2009).

[69] Lin A (2021). Minimum Reporting Standards for in vivo Magnetic Resonance Spectroscopy (MRSinMRS): Experts’ consensus recommendations. NMR Biomed.

[70] Smith SM, Nichols TE (2009). Threshold-free cluster enhancement: addressing problems of smoothing, threshold dependence and localisation in cluster inference. Neuroimage.

[71] Winkler AM, Ridgway GR, Webster MA, Smith SM, Nichols TE (2014). Permutation inference for the general linear model. Neuroimage.

[72] Alexander GE, DeLong MR, Strick PL (1986). Parallel organization of functionally segregated circuits linking basal ganglia and cortex. Annu Rev Neurosci.

[73] Benjamini Y, Hochberg Y (1995). Controlling the False Discovery Rate: A Practical and Powerful Approach to Multiple Testing. Journal of the Royal Statistical Society: Series B (Methodological).

[74] Kuhn, M., Jackson, S. & Cimentada, J. corrr: Correlations in R. (2020).

[75] RStudio Team. RStudio: Integrated Development Environment for R. (2020).

[76] Revelle, W. psych: Procedures for Psychological, Psychometric, and Personality Research. (2021).

[77] Wei, T. et al. corrplot: Visualization of a Correlation Matrix. (2021).

[78] Conigrave, J. corx: Create and Format Correlation Matrices. (2020).

[79] Wickham H (2019). Welcome to the tidyverse. Journal of Open Source Software.

[80] Wickham, H. & Bryan, J. readxl: Read Excel Files. (2019).

[81] Wickham, H. ggplot2: Elegant Graphics for Data Analysis. (2016).

[82] Wickham, H., François, R., Henry, L. & Müller, K. dplyr: A Grammar of Data Manipulation. (2021).

