# Supplementary material for: In Vivo 7-Tesla MRI Investigation of Brain Iron and Its Metabolic Correlates in Chronic Schizophrenia
Source: Schizophrenia (Heidelb). 2022 Oct 26;8(1):86. doi: 10.1038/s41537-022-00293-1 (PMC9605948; doi:10.1038/s41537-022-00293-1)
Supplement: Supplementary file 1 — Suplemental Material [file 41537_2022_293_MOESM1_ESM.docx]

**Supplementary Material**

***Between-group comparison of ROI volumes***

There were no significant between-group differences in the ROI volumes after FDR correction. In the schizophrenia group, increased volume in the left substantia nigra was observed (*p*=0.026), which did not survive FDR correction (Supplementary Table 1 and Supplementary Figure 1).

**Supplementary Table 1.** comparison of ROI volumes between groups

| ROI |  |  | control | schizophrenia | p-value |
| --- | --- | --- | --- | --- | --- |
| putamen | left | mean ± SD (mm^3^) | 5757.81 ± 611.45 | 5620.39 ± 512.83 | 0.834 |
|  | right | mean ± SD (mm3) | 5929.94 ± 590.30 | 5759.72 ± 486.83 | 0.667 |
| caudate nucleus | left | mean ± SD (mm3) | 4493.90 ± 441.84 | 4270.61 ± 372.49 | 0.297 |
|  | right | mean ± SD (mm3) | 4582.41 ± 458.82 | 4509.67 ± 365.36 | 0.965 |
| nucleus accumbens | left | mean ± SD (mm3) | 441.94 ± 55.95 | 415.65 ± 47.59 | 0.339 |
|  | right | mean ± SD (mm3) | 487.59 ± 65.93 | 455.94 ± 60.80 | 0.339 |
| globus pallidus | left | mean ± SD (mm3) | 1341.23 ± 134.08 | 1367.76 ± 157.35 | 0.291 |
|  | right | mean ± SD (mm3) | 1249.62 ± 130.59 | 1263.55 ± 159.67 | 0.326 |
| substantia nigra | left | mean ± SD (mm3) | 537.80 ± 87.92 | 588.83 ± 109.96 | **0.026** |
|  | right | mean ± SD (mm3) | 522.88 ± 94.16 | 546.68 ± 61.54 | 0.130 |
| thalamus | left | mean ± SD (mm3) | 6861.13 ± 713.76 | 6559.59 ± 693.18 | 0.511 |
|  | right | mean ± SD (mm3) | 6866.29 ± 677.39 | 6672.27 ± 677.48 | 0.893 |
| hippocampus | left | mean ± SD (mm3) | 3741.70 ± 447.50 | 3638.07 ± 292.35 | 0.711 |
|  | right | mean ± SD (mm3) | 3776.20 ± 473.61 | 3644.47 ± 316.67 | 0.650 |

ROI = region of interest, SD = standard deviation.

**Supplementary Figure 1.** Between-group comparison of ROI volumes. GP = globus pallidus, NAC = nucleus accumbens, SN = substantia nigra, TH = thalamus. Error bars indicate standard deviations.


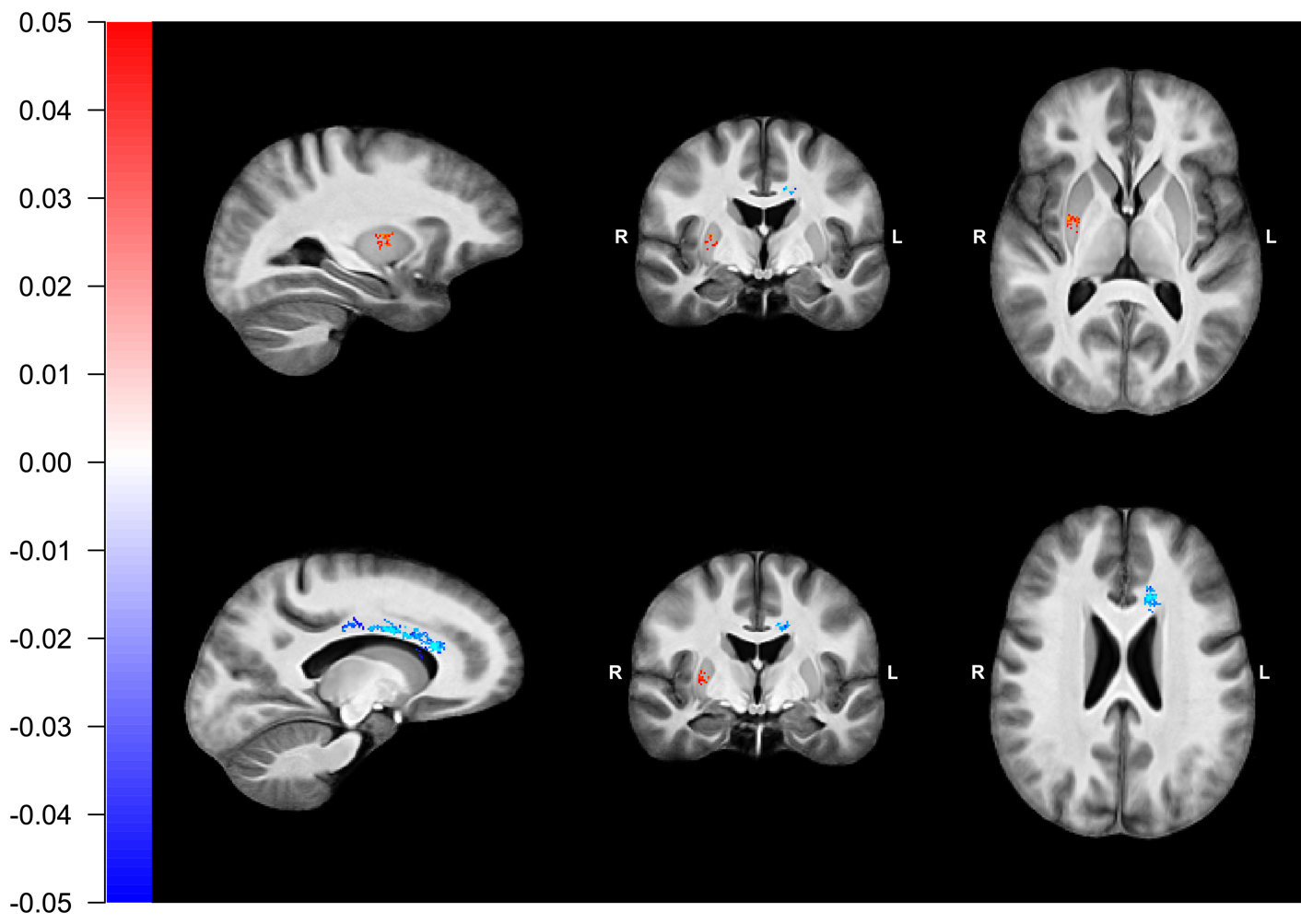


**Supplementary** **Figure 2.** Voxel-wise whole-brain comparison of QSM between groups with Threshold-Free Cluster Enhancment method. Clusters with higher and lower QSM in the schizophrenia group compared to controls are shown in red and blue respectively. Color coding corresponds to the p-values.

**
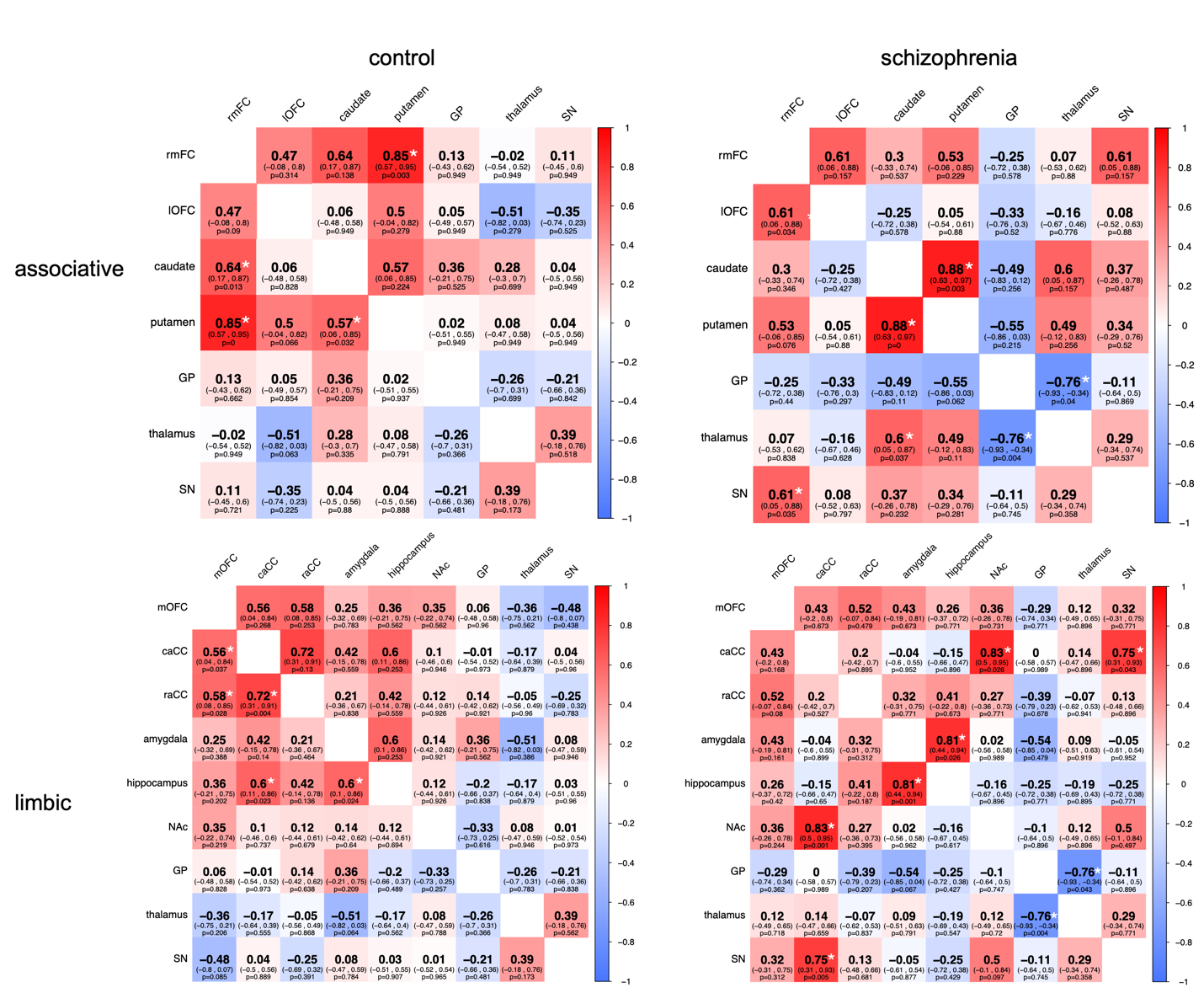
**

**Supplementary** **Figure 3.** Correlation matrix in QSM values between ROIs along the associative (top) and limbic (bottom) cortico-subcortical pathways in the control (left) and schizophrenia (right) groups. Numbers with larger and bold fonts are correlation coefficients, with 95% confidence interval (second line) and unadjusted p-values (third line) beneath them. In each matrix, p-values that are adjusted for multiple comparisons are shown the upper triangle and raw p-values are presented in the lower triangle. Asterisks indicate statistical significance. caCC = caudal anterior cingulate cortex, GP = globus pallidus, lOFC = lateral orbitofrontal cortex, mOFC = medial orbitofrontal cortex, NAc = nucleus accumbens, raCC = rostral anterior cingulate cortex, rmFC = rostral middle frontal cortex, SN = substantia nigra.

**
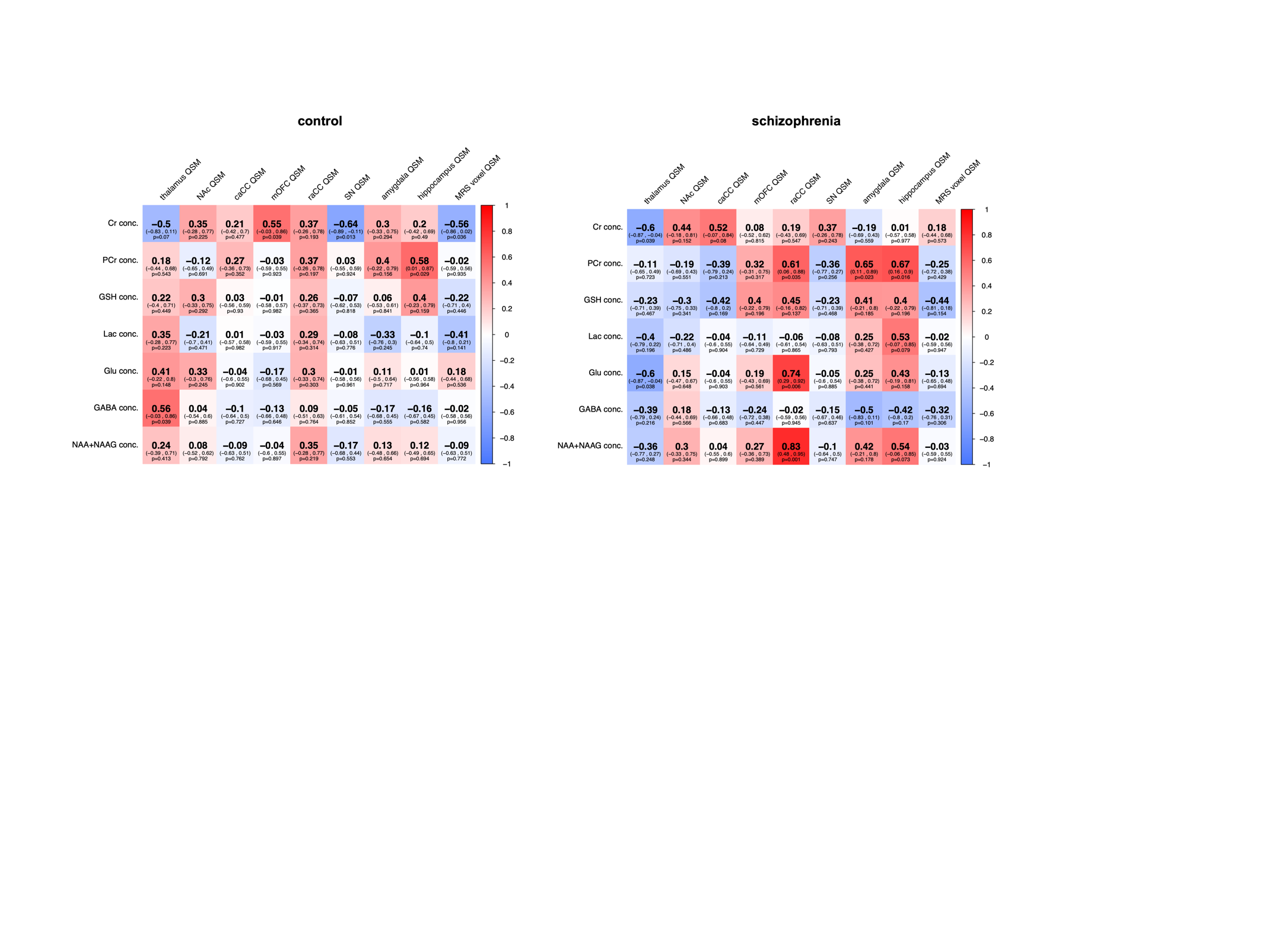
**

**Supplementary Figure 4.** Correlation between QSM in the limbic CSTC circuit structures and neurometabolites in the anterior cingulate cortex. Numbers with larger and bold fonts are correlation coefficients, with 95% confidence interval (second line) and unadjusted p-values (third line) beneath them. Asterisks indicate significant unadjusted p-values. caCC = caudal anterior cingulate cortex, conc. = concentration, Cr = creatine, GABA = gamma-aminobutyric acid, Glu = glutamate, GSH = glutathione, Lac = lactate, mOFC = medial orbitofrontal cortex, NAA = N-acetyl-aspartate, NAAG = N-acetyl-aspartyl-glutamate, NAc = nucleus accumbens, PCr = phosphocreatine, raCC = rostral anterior cingulate cortex, SN = substantia nigra


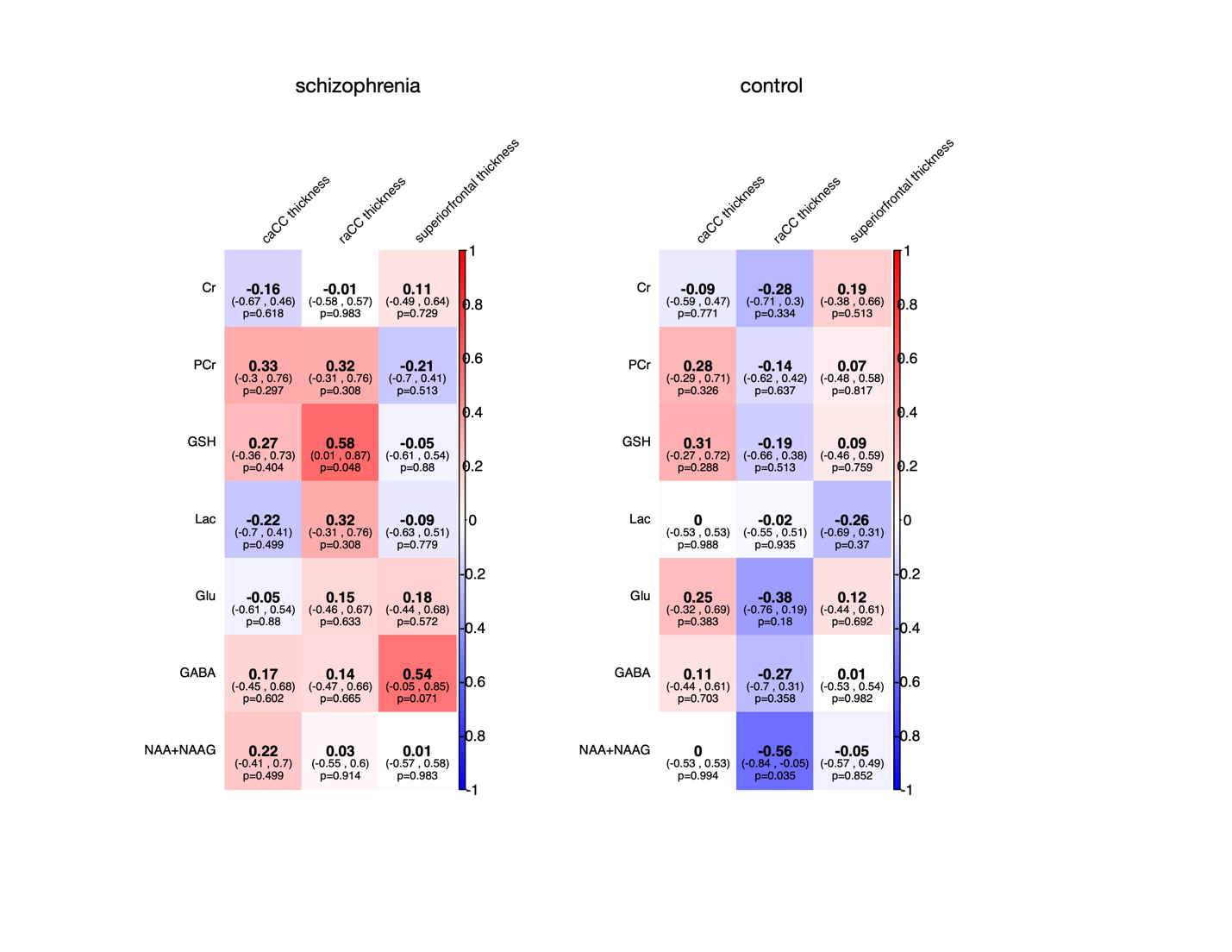


**Supplementary Figure 5.** Correlation between neurometabolites and cortical thickness. Numbers with larger and bold fonts are correlation coefficients, with 95% confidence interval (second line) and unadjusted p-values (third line) beneath them. Asterisks indicate significant unadjusted p-value. caCC = caudal anterior cingulate cortex, Cr = creatine, GABA = gamma-aminobutyric acid, Glu = glutamate, GSH = glutathione, Lac = lactate, NAA = N-acetyl-aspartate, NAAG = N-acetyl-aspartyl-glutamate, PCr = phosphocreatine, raCC = rostral anterior cingulate cortex.
